# Halo Phenomenon in Lobular Capillary Hemangioma: A Case Report of a Pyogenic Granuloma With Surrounding Cutaneous Hypopigmentation and Review of Tumors With Halo Phenomenon

**DOI:** 10.7759/cureus.43228

**Published:** 2023-08-09

**Authors:** Philip R Cohen, Nikolas Gutierrez, Christof P Erickson, Antoanella Calame

**Affiliations:** 1 Dermatology, University of California, Davis Medical Center, Sacramento, USA; 2 General Practice, United States Naval Mobile Construction Battalion 3, Port Hueneme, USA; 3 Dermatopathology, Compass Dermatopathology, San Diego, USA

**Keywords:** pyogenic, phenomenon, nevus, lobular, hypopigmentation, hemangioma, halo, granuloma, capillary, angioma

## Abstract

A halo phenomenon describes a skin neoplasm that is surrounded by a hypopigmented or white halo. Halo lesions have been observed in association with an epithelial neoplasm (seborrheic keratosis), a fibrous lesion (surgical scar), a keratinocyte malignancy (basal cell carcinoma), melanocytic neoplasms, and vascular lesions. Benign lesions (café au lait macules and nevi) and malignant tumors (primary and metastatic melanoma) are melanocytic neoplasms that have developed perilesional halos. Halo nevi are a commonly occurring manifestation of a halo phenomenon; however, perilesional hypopigmented halos have also been observed around nevi in patients following treatment with antineoplastic drugs, acquisition of COVID-19 (infection and vaccine), the occurrence of a visceral tumor (including not only melanoma, but also papillary thyroid carcinoma and neuroendocrine cancer of the lung), surgery (such as the excision of a primary melanoma), and Turner syndrome. A halo phenomenon has also been observed in patients with congenital (capillary malformation-arteriovenous malformation and congenital hemangioma) or acquired (angioma, eruptive pseudoangiomatosis, infantile hemangioma, and lobular capillary hemangioma) vascular lesions. In summary, a halo phenomenon can occur in association with primary lesions of various embryologic derivations. Most commonly, they have been observed in around nevi and vascular tumors. Halo lobular capillary hemangioma can be added to the list of acquired vascular lesions with the potential to develop a halo phenomenon. The preservation of melanocytes with loss of melanin pigment expression in the reported patient suggests the possibility that a post-inflammatory etiology may be responsible for the genesis of her halo lobular capillary hemangioma.

## Introduction

A halo phenomenon in a lesion often refers to annular hypopigmentation that surrounds the tumor. Leukoderma acquisitum centrifugum was previously used to describe the occurrence when the halo is hypopigmented. However, this terminology has not persisted in literature, and the observation of a cutaneous hypopigmented halo is more commonly designated as Sutton’s phenomenon [1-3]. In this paper, the halo phenomenon shall be used to describe the occurrence of a dermatosis or cutaneous neoplasm that is circumscribed by a hypopigmented or white halo of adjacent skin.

Lobular capillary hemangioma is a benign vascular lesion that can occur on the skin or mucosa. One of the designations originally used to describe the tumor was granuloma telangiectodes; yet, this terminology was subsequently replaced by pyogenic granuloma. However, since the neoplasm was neither infectious in etiology nor granulomatous in pathology, its name was changed to lobular capillary hemangioma [4,5].

A woman presented with an acquired red tumor that was surrounded by depigmented, white-appearing skin on her lower extremity. The clinical and pathological features of her lobular capillary hemangioma with halo phenomenon are discussed. In addition, other cutaneous neoplasms in which the halo phenomenon has been observed are reviewed [1-3,6-20].

## Case presentation

A healthy 50-year-old Caucasian woman with a history of seasonal allergies presented for evaluation of an asymptomatic red lesion on her distal right leg. She did not recall how long the lesion had been present but noted that it would occasionally become irritated. Indeed, it had previously spontaneously bled on two occasions.

She was a healthy woman. Her family history was significant for her father having had melanoma and several basal cell carcinomas. A mild compound dysplastic nevus had been excised from her left lower back 13 years earlier, and two irritated seborrheic keratoses had been removed from her left flank one month earlier.

**Figure 1:**
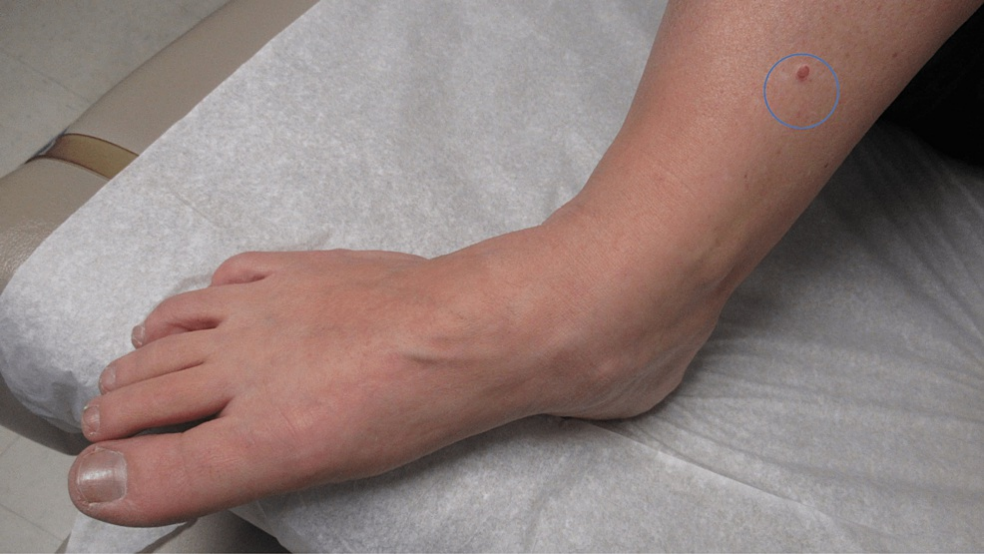
Clinical presentation of a halo lobular capillary hemangioma on the distal right leg of a 50-year-old Caucasian woman A 5.0 x 4.0-millimeter red nodule with a white epithelial collarette and surrounding asymmetric hypopigmented patch (within the blue oval) is located on her medial right leg proximal to the ankle. There were two prior episodes of the lesion spontaneously bleeding.

**Figure 2:**
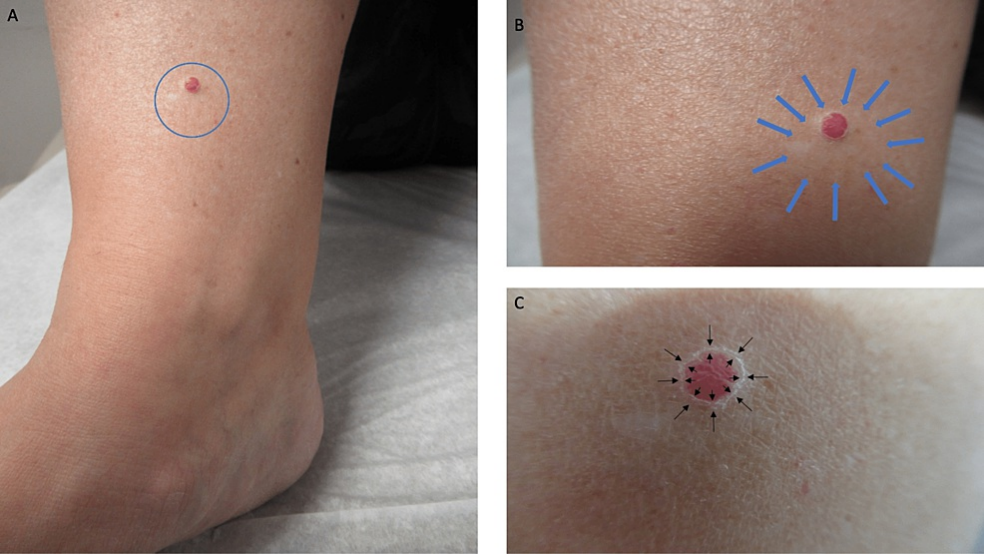
Morphologic appearance of a lobular capillary hemangioma with a perilesional hypopigmented patch A distant (A) view of the medial right lower extremity proximal to the ankle of a 50-year-old Caucasian woman (within the blue oval) shows a halo lobular capillary hemangioma. A closer view (B) shows an asymmetric hypopigmented patch that surrounds the lesion; its outline is demarcated by the blue arrows. Another closer view (C) shows superficial scaling overlying the erythematous central portion of the lesion and a white epithelial collarette surrounding the lesion; the raised edge of the collarette, at the edge of the vascular tumor, is present between the black arrows.

Cutaneous examination showed a non-tender, 5.0 x 4.0-millimeter erythematous nodule with superficial scaling and a white epithelial collarette on her distal medial right leg proximal to the ankle (Figure 1). A hypopigmented asymmetric halo of the skin surrounded the lesion. The skin adjacent to the perilesional halo was hyperpigmented (Figure 2).

A tangential excision, using the shave technique, was performed. The biopsy removed the entire lesion and some of the hypopigmented surrounding halo. The wound was allowed to heal by the second intention; the patient applied mupirocin ointment 0.1% three times daily.

**Figure 3:**
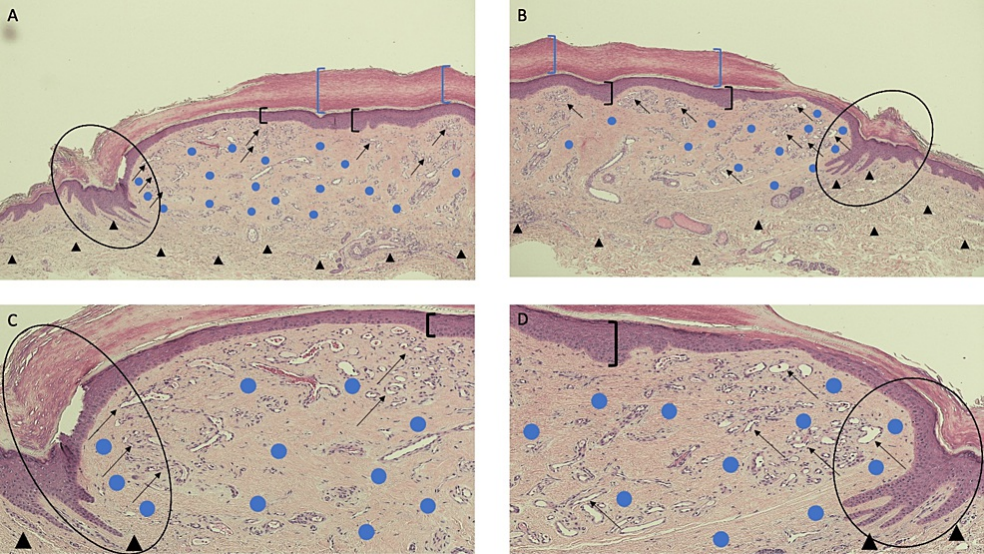
Lobular capillary hemangioma with halo phenomenon: hematoxylin and eosin-stained central portion of lesion and epidermal collarette Lower (A and B) and higher (C and D) magnification views show hyperkeratosis (within the blue brackets), acanthosis (within the black brackets), and a collarette of the epithelium (within the black oval). The nodular lesion shows lobules of vascular proliferation (black arrows) within a fibrotic dermal stoma (blue circles) with minimal inflammation. The dermis, both deep and lateral to the vascular tumor, has solar elastosis (black triangles) (hematoxylin and eosin stain: A, x10; B, x10; C, x20; D, x20).

**Figure 4:**
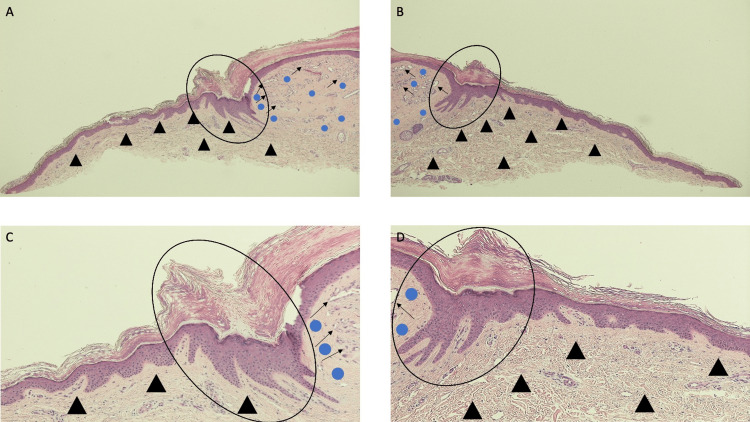
Lobular capillary hemangioma with perilesional halo: hematoxylin and eosin-stained tissue specimen of halo adjacent to the lesion, epidermal collarette, and lateral portion of the lesion Lower (A and B) and higher (C and D) magnification views show lobules of capillaries and small vessels (black arrows) and minimal accompanying inflammation within a fibrotic dermis (blue circles) in the lateral portion of the hemangioma. The epidermal collarette (within the black oval) surrounds and extends beneath the vascular tumor. There is solar elastosis (black triangles) in the lateral portion of the specimen where the hypopigmented perilesional was clinically observed (hematoxylin and eosin stain: A, x10; B, x10; C, x20; D, x20).

Microscopic examination of hematoxylin and eosin-stained sections showed a nodular lesion (Figure 3). The central portion of the polypoid nodule demonstrated a proliferation of capillaries, surrounded by fibrotic stroma in the dermis; only a minimal inflammatory cell infiltrate was present (Figure 4). The epidermis overlying this portion of the lesion was acanthotic with compact orthokeratosis.

**Figure 5:**
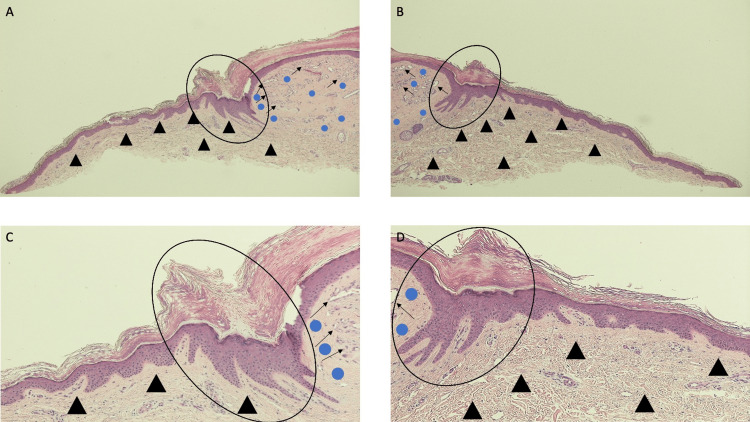
Lobular capillary hemangioma with perilesional halo: hematoxylin and eosin-stained tissue specimen of halo adjacent to the lesion, epidermal collarette, and lateral portion of the lesion Lower (A and B) and higher (C and D) magnification views show lobules of capillaries and small vessels (black arrows) and minimal accompanying inflammation within a fibrotic dermis (blue circles) in the lateral portion of the hemangioma. The epidermal collarette (within the black oval) surrounds and extends beneath the vascular tumor. There is solar elastosis (black triangles) in the lateral portion of the specimen where the hypopigmented perilesional was clinically observed (hematoxylin and eosin stain: A, x10; B, x10; C, x20; D, x20).

The lateral portion of the lesion shows a collarette of epithelium surrounding the vascular tumor (Figure 5). Mounds of orthokeratosis form the white scaling noted clinically. Elongated epithelial rete ridges extend into the dermis separating the dermal stroma of the tumor from the adjacent and underlying dermis. Solar elastosis is present in the dermis adjacent to the tumor.

**Figure 6:**
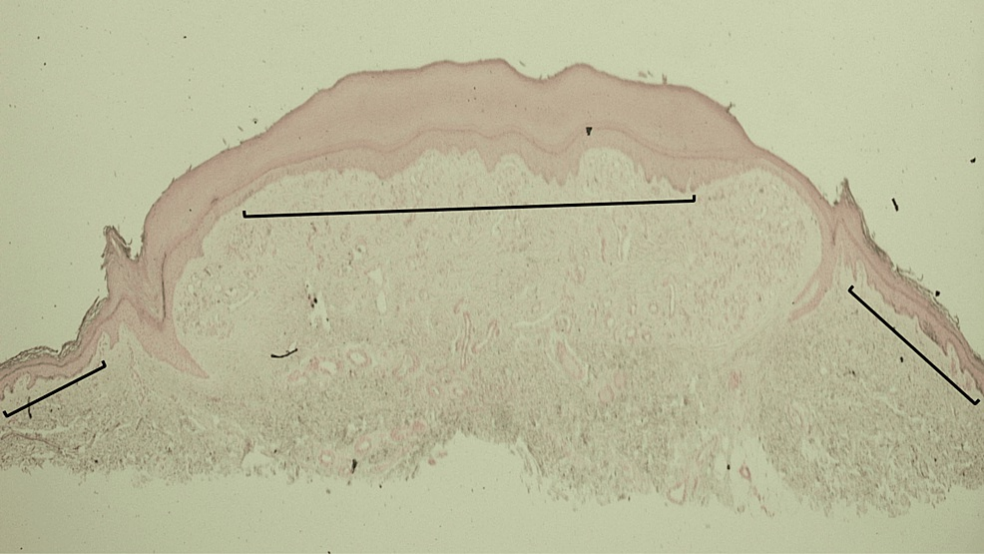
Microscopic examination of the Fontana-Masson-stained halo lobular capillary hemangioma on a 50-year-old woman’s distal medial right leg The low magnification view of the lobular capillary hemangioma with a perilesional halo shows positive staining with a Fontana-Masson stain in the central portion of the vascular tumor and lateral to the epidermal collarette on both sides of the specimen (cells in the basal layer of the epidermis, found within the black brackets). The Fontana-Masson stain demonstrates the presence of melanin; therefore, the melanin is not shown to be present in the epidermal collarette and the skin, medially and laterally, adjacent to the epidermal collarette on both sides of the specimen (Fontana-Masson stain: x 2).

**Figure 7:**
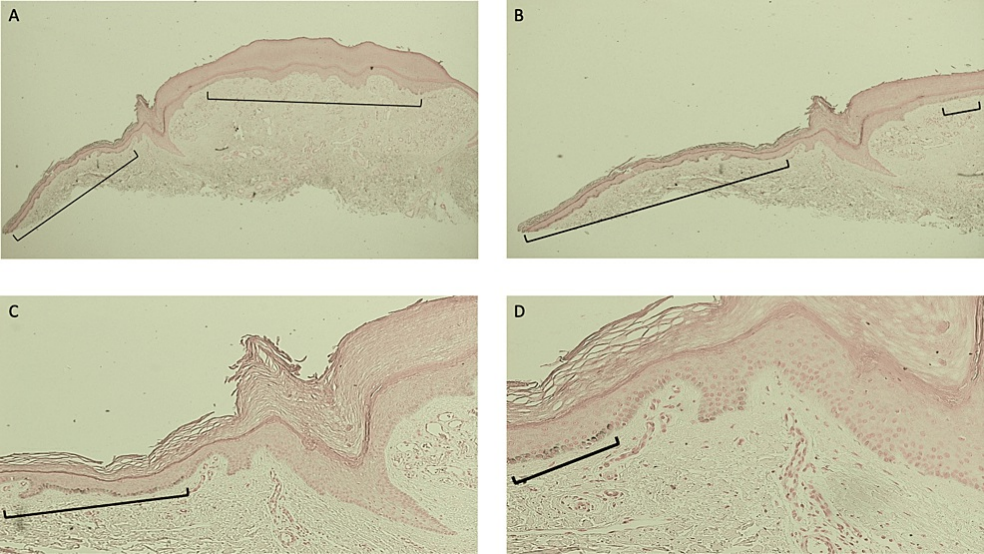
Lobular capillary hemangioma with halo phenomenon: Fontana-Masson-stained left side of the lesion and perilesional halo Low (A) and higher (B, C, and D) magnification views of the left side of the Fontana-Masson-stained halo lobular capillary hemangioma do not show melanin in the areas corresponding to the epidermal collarette and the adjacent skin--both medial and lateral to the collarette. Melanin is present, as demonstrated by the positive staining of cells in the basal layer of the epidermis (within the black brackets) at the edges of the specimen and in the center of the vascular lesion (Fontana-Masson stain: A, x4; B, x10; C, x20; D, x40).

**Figure 8:**
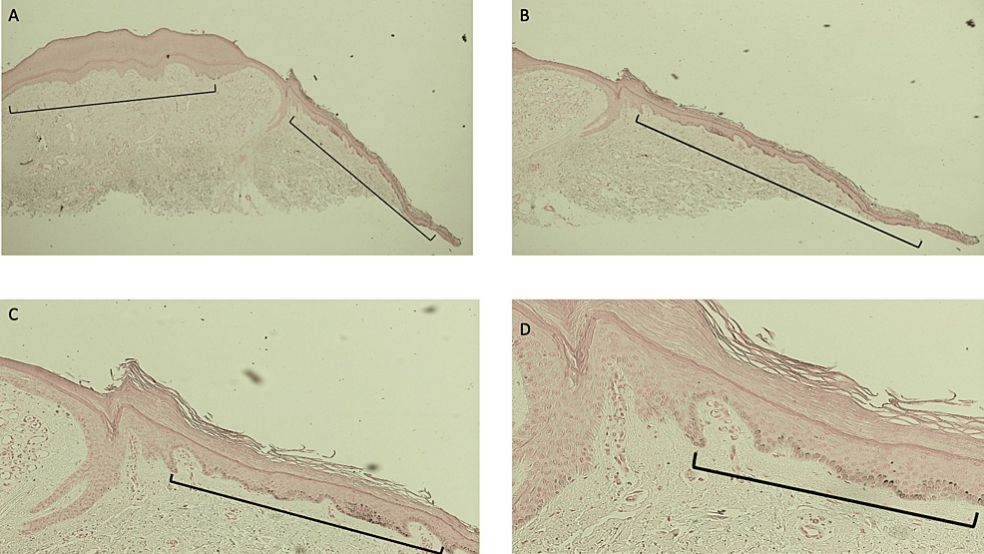
Lobular capillary hemangioma with perilesional halo: right side of the lesion and adjacent halo stained with Fontana-Masson stain Low magnification (A) and higher magnification (B, C, and D) views of the right side of the halo lobular capillary hemangioma stained with the Fontana-Masson stain. Melanin is present in the basal layer cells found in the center of the vascular tumor and in the lateral portion of the specimen (denoted by the black staining of the cells in the basal layer of the epidermis within the black brackets). Melanin expression is absent in the areas of the specimen that show the epidermal collarette (which appeared white) and the adjacent skin (both medial and lateral) to the collarette; the absence of melanin adjacent to the central lesion clinically correlates with the hypopigmented halo that surrounds the vascular tumor (Fontana-Masson stain: A, x4; B, x10; C, x20; D, x40).

The Fontana-Masson stain is used to detect melanin pigment. Sections stained with the Fontana-Masson stain showed positive staining in the central portion of the vascular tumor, overlying the area of vascular proliferation (Figures 6-8). However, an absence of epidermal basal cell layer staining was noted not only in the area of the epidermal collarette but also both medial and lateral to the epidermal collarette; this absence of Fontana-Masson stain corresponds to the location of the clinical perilesional depigmented halo.

**Figure 9:**
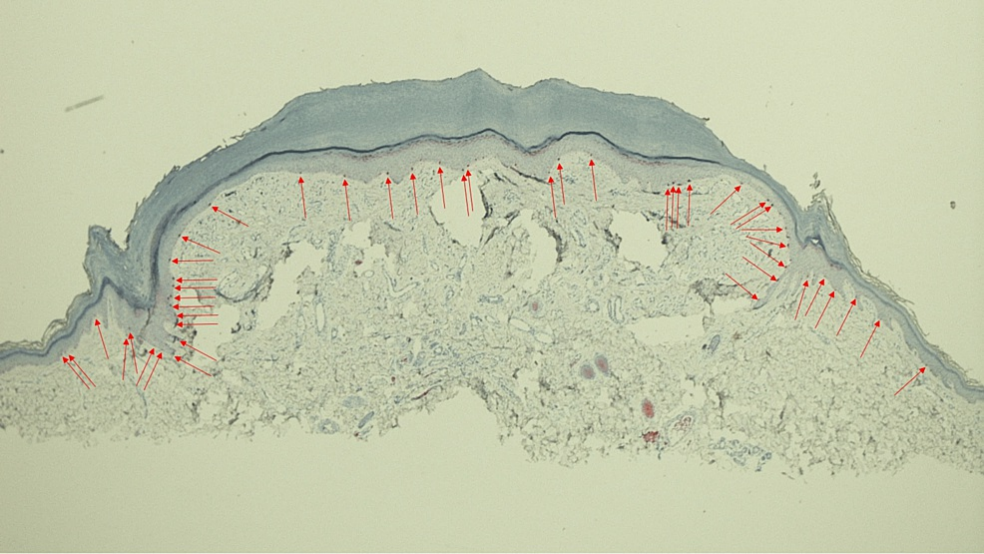
Microscopic examination of a 50-year-old woman’s distal medial right leg halo lobular capillary hemangioma stained with melanoma antigen recognized by the T-cell (MART-1) stain Low magnification view of lobular capillary hemangioma with perilesional halo after staining with melanoma antigen recognized by the T-cell (MART-1) stain, using a red chromogen to demonstrate the positive staining cells, shows individual melanocytes (red arrows) present within the basal layer of the epidermis. The melanocytes are uniformly present not only within the central portion of the vascular tumor but also in the epidermis of the epidermal collarette (which clinically appeared white) and the skin adjacent to the collarette (which clinically presented as a halo surrounding the vascular tumor) (MART-1 stain: x2).

**Figure 10:**
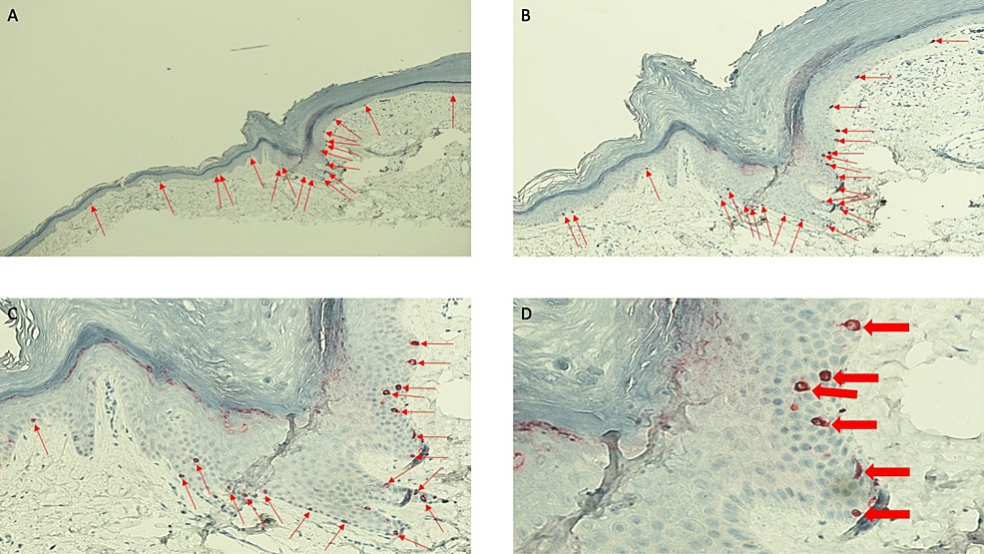
Lobular capillary hemangioma with a halo phenomenon: melanoma antigen recognized by the T-cell (MART-1)-stained left side of the lesion and perilesional halo Low (A) and higher (B, C, and D) magnification views of the left side of the melanoma antigen recognized by the T-cell (MART-1)-stained halo lobular capillary hemangioma shows red staining cells predominantly in the basal layers of the epidermis (red arrows). MART-1 is an immunoperoxidase stain that demonstrates melanocytes; a red chromogen identifies the positive staining cells. The entire specimen, including the central vascular tumor, the epidermal collarette, and the perilesional tissue, all contain melanocytes that are evenly distributed along the epidermal basal layers (MART-1 stain: A, x4; B, x10; C, x20; D, x40).

**Figure 11:**
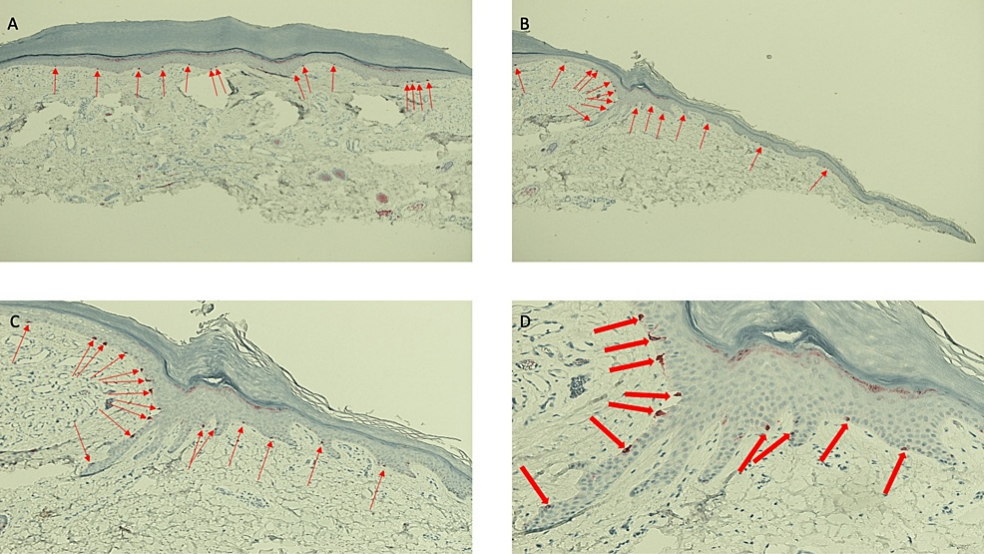
Lobular capillary hemangioma with perilesional halo: right side of the lesion and adjacent halo stained with melanoma antigen recognized by the T-cell (MART-1) stain Low magnification (A) and higher magnification (B, C, and D) views of the right side of the halo lobular capillary hemangioma stained with melanoma antigen recognized by the T-cell (MART-1) stain. Melanocytes are present in the basal layer cells of the vascular tumor and in the lateral portion of the specimen (denoted by the red arrows pointing to the red-staining cells). In contrast to an autoimmune disorder such as vitiligo in which there is both melanin expression and melanocytes are diminished, the halo lobular capillary hemangioma demonstrates the loss of pigmentation but the preservation of melanocytes in the epidermal basal cells that are located in the hypopigmented perilesional halo; this finding is consistent with a post-inflammatory process (MART-1 stain: A, x4; B, x10; C, x20; D, x40).

The melanoma antigen recognized by the T-cell (MART-1) stain is used to detect melanocytes. Sections stained with the MART-1 stain showed uniform positive staining along the epidermal basal cell layer of the entire lesion and the epidermis adjacent to the lesion (Figures 9-11). Melanocytes are uniformly present within the epidermal collarette (which clinically appeared white) and in the skin surrounding the lesion (which clinically appeared hypopigmented).

The pathologic findings observed on the hematoxylin and eosin-stained sections are those of lobular capillary hemangioma. However, the correlation of the clinical presentation of the perilesional hypopigmented halo also included the white-appearing epidermal collarette surrounding the central vascular lesion established the diagnosis of a halo lobular capillary hemangioma. The Fontana-Masson stain and MART-1 stain, demonstrating the loss of epidermal basal cell layer pigmentation but the preservation of melanocytes in the hypopigmented halo, are suggestive of a post-inflammatory process instead of an autoimmune disorder (such as vitiligo in which there is both melanin expression and melanocytes are diminished).

## Discussion

The halo phenomenon is observed in several conditions that affect the skin. Some of these cutaneous disorders include granulomatous diseases (such as sarcoidosis), infection-related non-specific lesions (such as syphilis), and papulosquamous diseases (such as lichen planus and psoriasis) [3]. In addition, benign and malignant lesions have been described that demonstrate the halo phenomenon (Table 1) [1-3,6-20].

**Table 1:** Lesion-associated halo phenomenon ^a^This includes a seborrheic keratosis, a benign epithelial neoplasm. ^b^This includes not only the scar that is located at the site of a prior melanoma but also the surgical scar associated with the locoregional nodal dissection or cutaneous metastasis excision; in all circumstances, scleroatrophic alterations (suggestive of postsurgical scleroatrophy) were absent. ^c^This includes basal cell carcinoma. ^d^This also includes the lobular capillary hemangioma in the current report.

Lesion	References
Epithelial neoplasm^a^	[1]
Fibrous lesion^b^	[6]
Keratinocyte malignancy^c^	[3]
Melanocytic neoplasm	[2,7-12]
Vascular lesion^d^	[13-20]

A seborrheic keratosis is a benign epithelial neoplasm; at least three individuals with halo seborrheic keratoses have been described. One of the elderly patients was a man with numerous seborrheic keratoses that had been present for 20 years; a depigmented halo surrounded five of the lesions. Another man’s appearance of whitish halos around his seborrheic keratoses coincided with the diagnosis of colon cancer; following colon surgery, all the halos disappeared [1].

Peri-cicatricial hypomelanotic macules have been observed to develop around scars in melanoma patients; in contrast to postsurgical scleroatrophy, scleroatrophic alteration is not present in the cicatrix with surrounding hypopigmentation. The halo phenomenon has localized around the surgical scars in three specific areas: the primary melanoma excision site, the excision sites of cutaneous melanoma metastases, and the scar associated with the locoregional nodal dissection. In a study of 2954 melanoma patients, the peri-cicatricial halo phenomenon was observed around the melanoma surgical scar of 17 individuals [6].

The halo phenomenon has been described, albeit rarely, in association with basal cell carcinoma. The initial patient was reported as having basal cell carcinoma with annular leukoderma mimicking leukoderma acquisitum centrifugum in a 57-year-old man whose tumor was on his posterior neck. Subsequently, additional patients with halo basal cell carcinoma have been observed [3].

Melanocytic neoplasms that can be associated with the halo phenomenon include benign lesions and malignant tumors (Table 2) [2,6-12]. The benign lesions are café au lait macules and nevi [2,6-12]. The malignant tumors include primary malignant melanoma and cutaneous melanoma metastases [2,6].

**Table 2:** Melanocytic neoplasm-associated halo phenomenon BRAF: v-raf murine sarcoma a viral oncogene homolog B1, ERK: extracellular protein kinase, MAPK: mitogen-activated protein kinase, MEK: MAPK/ERK kinase ^a^This lesion is also referred to as Sutton nevus. Commonly, the nevus is either compound or junctional; however, the halo phenomenon has occurred with other types of melanocytic nevi including atypical nevi, congenital giant nevocellular nevi, congenital melanocytic nevi, and Spitz nevi. ^b^Halo phenomenon has occurred after patients with melanoma or other visceral tumors have been systemically treated with either immunotherapy (such as atezolizumab, interferon beta, ipilimumab, nivolumab, and pembrolizumab) or targeted therapy with BRAF (is a serine/threonine kinase; it is a human gene that encodes a protein B-Raf) and MEK inhibitors (such as dabrafenib and trametinib). Furthermore, in patients with multiple sclerosis, the halo phenomenon has also been associated with interferon beta treatment. ^c^A healthy 35-year-old man, who was not taking any medications, developed a sudden eruption of 35 halo nevi predominantly located on his trunk four weeks after he received the second dose of COVID-19 vaccine, which was four weeks after he received the first dose [12]. Eruptive halo nevi have been observed in other individuals either after receiving the COVID-19 vaccine or experiencing COVID-19 infection. ^d^The appearance of multiple halo nevi has been associated with the presence or development of the following cancers: melanoma, papillary thyroid carcinoma, and neuroendocrine (atypical carcinoid) tumor of the lung. ^e^Halo nevi developed after the excision of primary melanoma.

Neoplasm	References
Benign lesion	[2,7-12]
Café au lait macules	[9]
Nevus	[2,6-8,10-12]
Halo^a^	[2,6,10,11]
Drug (antineoplastic)-induced^b^	[7,8]
COVID-19^c^	[12]
Malignancy-associated^d^	[6,12]
Post surgical^e^	[7]
Turner syndrome	[12]
Malignant melanoma	[2,6]
Primary	[2]
Metastatic	[2,6]

A halo nevus, also known as Sutton nevus, clinically appears as a zone of hypopigmentation surrounding a centrally located pigmented nevus. In a study of 124 cases, dysplastic nevi (48, 38.7%), benign acquired (junctional or compound) nevi (44, 35.5%), and nevi with congenital features (25, 20.2%) were the most commonly associated lesions. However, melanoma (three, 2.4%), regressed nevus (two, 1.6%), Spitz nevus (one, 0.8%), and combined (congenital and blue) nevus (one, 0.8%) were the other pigmented lesions observed [10,11].

Idiopathic halo nevi have been observed in approximately 1% of the population. They usually appear in children, at a mean onset age of 15 years; indeed, a study including 124 nevi noted that the lesions initially occurred in adolescents from 13 to 19 years old (39, 31.5%) and young adults from 20 to 35 years old (34, 27.5%). Therefore, the new onset of halo nevi in an older adult may be cause for concern [10,11].

The back is the most frequent location of halo nevi. The trunk, including the back (59, 47.6%), chest (25, 20.2%), and abdomen (7, 5.6%), were the sites of 91 (73.4%) of these nevi in a study of 124 lesions. They have also been observed on the extremities and the head and neck [10,11].

The clinical presentation of halo nevi is classified into four stages based on the evolution of the lesion: a central nevus with a surrounding depigmented rim (stage I), pigment loss within the central nevus which is surrounded by a depigmented rim (stage II), total nevus disappearance and a residual round patch, including the site of the prior nevus and the surrounding rim, of depigmentation (stage III), and repigmentation of the site to the color of the adjacent, normal-appearing skin (stage IV). Microscopic features of halo nevus typically consist of not only the primary melanocytic lesion but also an accompanying, often dense, inflammatory infiltrate of lymphocytes that can fill the papillary dermis and extend into the underlying reticular dermis. As the halo nevus becomes older, there can be a marked decrease or complete absence of the melanocytic lesion or the inflammatory infiltrate, or both [2,11].

The pathogenesis of halo nevi remains to be established; there are two theories. Less favored is the antibody theory since activated and cell-proliferating lymphocytes, after excision of the halo nevus, disappear. The second theory is related to cytotoxic T-cell response; the T-cell lymphocytes (which comprise about 80% of the infiltrate), have cytotoxic activity as demonstrated by Fas ligand, granzyme B, and perforin positivity [11].

The eruptive onset of halo nevi has also been associated with antineoplastic drugs, coronavirus vaccine and infection, visceral cancers, surgery, and Turner syndrome. Angiokeratomas, hemangiomas, hirsutism, and halo nevi are cutaneous manifestations of Turner syndrome. Indeed, compared to vitiligo, there is an increased prevalence of halo nevi in patients with Turner syndrome [12].

In patients with melanoma, the administration of not only immunotherapy (such as ipilimumab, nivolumab, and pembrolizumab) but also targeted therapy with v-raf murine sarcoma a viral oncogene homolog B1 (BRAF) and mitogen-activated protein kinase (MAPK)/extracellular protein kinase (ERK) kinases (MEK), such as dabrafenib and trametinib. Immunotherapy using atezolizumab was also associated with the onset of halo nevi in a patient with lung cancer. In addition, the appearance of halo nevi has been observed in patients with multiple sclerosis receiving interferon beta immunotherapy [7,8].

The virus-related onset of eruptive halo nevi has also been reported. In several patients, receiving a COVID-19 vaccine has been associated with the development of halo nevi. In addition, the new appearance of multiple halo nevi has also been temporally correlated to the occurrence of COVID-19 infection [12].

The new onset of halo nevi has been observed in melanoma patients. A study of 16 consecutive patients with eruptive halo nevi revealed not only melanoma in six individuals but also other visceral tumors in three of the patients. The patients with melanoma either had melanoma in situ (two patients), malignant melanoma of the skin (two patients), melanoma skin metastases without a known primary tumor (one patient), or primary cutaneous melanoma with pulmonary metastases (one patient). The other cancers included papillary thyroid carcinoma (two patients) and neuroendocrine (atypical carcinoid) tumors of the lung (one patient) [7,11,12].

The development of halo nevi following the excision of a primary cutaneous malignant melanoma has also been observed; specifically, either a single (two patients) or multiple (three patients) halo nevi appeared after the individual had a melanoma surgically removed. In three of the patients, the halo nevi developed within one (one patient) or two (two patients) weeks after the melanoma was excised. The development of multiple halo nevi in another patient prompted the excision of a warty plantar tumor which was a melanoma. The interval of time between melanoma removal and the appearance of the halo nevus was not provided for one patient [7].

Café au lait macules are benign flat pigmented macules or patches. The presence of six or more café au lait macules is one of the criteria for establishing the diagnosis of neurofibromatosis type 1 (von Recklinghausen disease). In Asian infants, congenital dermal melanocytosis (which is also referred to as a Mongolian spot is commonly observed on the back and buttocks. Therefore, in Asian infants with neurofibromatosis type 1, both café au lait macules and congenital dermal melanocytosis can be present [9].

A study evaluated a group of 95 children with neurofibromatosis type 1; 24 patients had at least one café au lait macule overlapping a congenital dermal melanocytosis on the back or buttock. The halo phenomenon of the café au lait macules was present in 21 children. The halo phenomenon was present for all the café au lait macules within the congenital dermal melanocytosis for 10 of the children; in contrast, the halo phenomenon was not present around all the café au lait macules of the other 11 children. Since three children with only one café au lait macule within or overlapping a congenital dermal melanocytosis did not show the halo phenomenon, the investigators proposed that the development of a halo café au lait macule might be related to the number of café au lait macules overlapping lesions of congenital dermal melanocytosis [9].

Primary malignant melanoma and melanoma cutaneous metastases are malignant melanocytic tumors that have been associated with the halo phenomenon. The diagnosis of halo melanoma may be discovered during the evaluation of the biopsy specimen of a suspected benign nevus with a halo phenomenon or it might be entertained during the initial evaluation of the pigmented lesion with a surrounding hypopigmented halo. For example, in the microscopic evaluation of 124 suspected halo nevi, 121 of the associated melanocytic lesions were benign; however, three of the pigmented lesions with surrounding depigmented halo were primary melanomas [10].

A pigmented lesion on the shoulder of a nine-year-old girl enlarged and, within four months, developed a surrounding zone of achromia. A complete examination revealed axillary lymphadenopathy and hepatosplenomegaly; pulmonary metastases subsequently developed. She died; the skin lesion and lymph node metastases were interpreted to be malignant melanoma [2].

The halo phenomenon also occurs surrounding melanoma cutaneous metastases. In a study of 2954 melanoma patients, localized vitiligo, which included not only peri-cicatricial hypopigmented lesions (17 patients) and halo nevi (six patients) but also focal hypopigmentation (21 patients), or generalized vitiligo (39 patients) was observed in 83 individuals. Four of the 21 patients with focal vitiligo had hypomelanotic lesions that developed around their cutaneous metastases [6].

A primary melanoma was excised from the back of a 70-year-old man. Within seven months, axillary metastases were discovered; three months later, multiple cutaneous metastases appeared as light blue nodules on his trunk and thigh. He received six months of chemotherapy; however, zones of leukoderma around several of the blue cutaneous metastases appeared six months after treatment has finished. New white halos continued to develop around the cutaneous metastases during the subsequent eight months. He died two years and seven months after his melanoma diagnosis; he had widespread visceral metastases not only to his skin but also to his heart, liver, pituitary, spleen, stomach, and testes [2].

Several benign vascular lesions have been associated with the halo phenomenon (Table 3) [13-20]. These include both congenital (capillary malformation-arteriovenous malformation and hemangioma) and infantile (hemangioma) vascular lesions. They also include acquired lesions such as an angioma, lobular capillary hemangioma, and eruptive pseudoangiomatosis.

**Table 3:** Vascular lesion-associated halo phenomenon CM-AVM: capillary malformation-arteriovenous malformation, CR: current report ^a^These are also referred to as Campbell de Morgan spots, cherry angioma, cherry hemangioma, hemangioma, senile angioma, and senile hemangioma. ^b^These are also referred to as granuloma telangiectodes and pyogenic granuloma.

Lesion	References
Angioma^a^	[12,13,14]
CM-AVM	[17]
Congenital hemangioma	[19]
Eruptive pseudoangiomatosis	[15,16]
Infantile hemangioma	[20]
Lobular capillary hemangioma^b^	[CR]

The capillary malformation is a congenital vascular malformation. It consists of dermal capillaries or venules that are increased in number or dilated or both. The vascular structures form a slow-flow, flat, pink-purple stain; as the child grows, the vascular malformation typically grows proportionately. Capillary malformations can occur as an isolated lesion; however, they can also be a component of various congenital syndromes [16].

Capillary malformation-arteriovenous malformation is an autosomal dominant condition. Heterozygous mutations in RAS p21 protein activator 1 (RASA1), located on chromosome 5q13.3, are associated with this vascular malformation. Affected individuals with the condition have atypical capillary malformation associated with arteriovenous malformations, arteriovenous fistulas, or Parkes Weber syndrome; the latter is characterized by a large cutaneous vascular stain with subcutaneous and intramuscular arteriovenous fistulas and overgrowth of the affected limb [16].

A clinical review of 45 patients with capillary malformation-arteriovenous malformation showed that all the patients had well-defined, round to oval, pink-purple or reddish-brown macules; these were located, in order of decreasing frequency, on the trunk (86%), upper extremities (71%), head and neck (64%), lower extremities (55%), palms (38%), oral mucosa (13%), soles (9%), and nasal mucosa (2%). The macules were surrounded by a white halo in 26 patients. In addition, pinpoint bright red macules with a surrounding pale halo were observed in nine patients; these were predominantly found on the face, forearms, and dorsum of hands [16].

Congenital hemangioma is a vascular lesion that is fully developed and present at birth. It typically appears as a violaceous plaque or nodule with surrounding pale halo and telangiectasias. However, the surrounding pale pallor can be absent or its color can be grayish [5,17].

After birth, a congenital hemangioma does not have a proliferative phase. However, spontaneous involution of a congenital hemangioma may occur; the observed clinical progression has been used to classify the lesion. The vascular tumor can be a rapidly involuting congenital hemangioma (RICH), a non-involuting congenital hemangioma (NICH), or a partially involuting congenital hemangioma (PICH) [5,17].

A retrospective study of 10 congenital hemangiomas, consisting of six RICH, three NICH, and one PICH, was performed. One of the RICH and the PICH both developed a residual anemic halo at the site of the vascular tumor during their regression [17].

Congenital hemangiomas have somatic activating mutations in GNAQ and GNA11. They also express vascular markers such as CD31 and CD34. However, they do not express lymphatic vessel markers such as podoplanin (D2-40) and lymphatic vessel endothelial receptor 1 (LYVE-1). In addition, they also do not express glucose transport-1 (GLUT-1) [5].

Patients with RICH can develop vascular lesion-associated complications such as coagulopathy, high-output cardiac failure, and thrombocytopenia; therefore, treatment of their tumor may be necessary. In contrast, systemic complications do not usually occur in patients with PICH and NICH. Therefore, these latter types of congenital hemangiomas do not require further treatment or can be surgically resected [5].

An infantile hemangioma is a GLUT-1 positive vascular lesion that usually appears one to two weeks after birth. It occurs in approximately 5% to 10% of all babies, making it the most common benign vascular tumor in infants. Clinically, it presents as an erythematous papule or nodule or plaque surrounded by a hypopigmented halo on the head and neck, followed by the trunk and the extremities; the lesion initially grows rapidly (during the first four months of age) and by five to seven months has stabilized in size [5,20].

Less commonly, an infantile hemangioma is present at birth. In this setting, the lesion may appear as a pale patch; often, within the pale area, thread-like telangiectasias are present. This precursor patch of infantile hemangioma has been referred to as the pallor sign [20].

In addition to the tumor expressing GLUT-1, infantile hemangioma not only demonstrate positivity for vascular markers such as CD31 and CD34 but also placenta-related biomarkers such as FcgammaRII, Lewis Y antigen, and merosin. They do not express lymphatic vessel markers such as D2-40 and LYVE-1 [5].

Prior to the child reaching the age of five years, many infantile hemangiomas have spontaneously regressed or involuted. However, complete regression may take up to ten years. Beta-blockers applied topically or administered orally or both, such as timolol or propranolol, may be used to treat infantile hemangiomas that have not spontaneously resolved [5].

There are several subtypes of infantile hemangiomas: focal, indeterminate, multifocal, and segmental. The latter, segmental infantile hemangiomas, occur in patients with posterior fossa anomalies, hemangioma, arterial anomalies, cardiac anomalies, and eye anomalies (PHACES) syndrome and lower body infantile hemangioma, urogenital anomalies, ulceration, myelopathy, bony deformities, anorectal malformations, arterial anomalies, and renal anomalies (LUMBAR) syndrome. In addition, although considered to be benign, infantile hemangiomas have a 24% complication rate; adverse events associated with infantile hemangiomas include disfigurement, functional impairment, and ulceration [5].

Angiomas are benign acquired vascular lesions; they are also referred to as Campbell de Morgan spots, cherry angioma, cherry hemangioma, hemangioma, senile angioma, and senile hemangioma. In affected individuals, they typically appear in the third decade of life. They present as small red papules, often less than four millimeters in diameter [13,14].

Halo angioma is the development of a depigmented to white halo around an angioma. There are only a small number of reports in the literature. However, the actual occurrence of the halo phenomenon around an angioma may be more prevalent than the paucity of published descriptions implies [13,14].

A study of angiomas was performed by inspecting the trunks of 488 patients, aged 40 years or older, who had at least one angioma. The investigator found that 5.1% (25 of 488) of the patients had at least one halo angioma; indeed, the halo phenomenon was discovered in 2% (69/3479) of the angiomas. Halo angiomas were more prevalent in patients aged 60 years and older, in individuals who had more than four angiomas, and in people whose angiomas were more than three millimeters in diameter [13].

Rarely, halo formation around an angioma has been associated with a systemic disease. A 54-year-old man, with idiopathic lobular panniculitis that had been treated continuously with tocilizumab for four years, presented with persistent widespread yellow macules and halo angioma of one-year duration. His work-up revealed diffuse plane normolipemic xanthomatosis associated with monoclonal gammopathy [15].

Lobular capillary hemangioma, also known as granuloma telangiectodes or pyogenic granuloma, is a benign vascular tumor. Similar to congenital hemangioma, it demonstrates positivity for CD31 and CD34 and does not express GLUT-1. It clinically appears as an erythematous, possibly pedunculated, papule that is surrounded by a collarette of the epithelium; the surface of the lesion may be ulcerated and bleeding may occur spontaneously or after minor trauma [4,5].

Pathologic features of a lobular capillary hemangioma demonstrate a proliferation of small blood vessels and capillaries, arranged in distinct lobules, in the dermis. In the central aspect of the lobules, a larger (feeding) vessel may be present. The surrounding stroma can be edematous or fibrotic or myxoid [4,5].

A dense infiltrate of neutrophils is often present throughout the dermis; this may be especially prominent if the lesion is ulcerated. However, in older lesions, the inflammatory infiltrate may be diminished or absent. Indeed, no inflammation was observed in the dermis of the lobular capillary hemangioma of the woman described in this report [4,5].

The overlying epidermis of a lobular capillary hemangioma may be intact or atrophic and ulcerated. The collarette of epithelium observed clinically can be observed in the lateral portion of the lesion. Elongated epidermal rete ridges extend into the dermis and surround the vascular lesion [4,5].

The halo phenomenon of a lobular capillary hemangioma, to the best of my knowledge, has not previously been described. The woman in this was not aware of the hypopigmented annular ring that surrounded the lesion on her leg. The skin adjacent to the halo lobular capillary hemangioma was hyperpigmented secondary to chronic sun exposure; the solar elastosis in the lateral aspects of the biopsy specimen from her leg correlates with her clinical history of extensive exposure to ultraviolet radiation.

Clinically, the white halo surrounding the woman’s lobular capillary hemangioma included the epidermal collarette and extended into the adjacent skin. The Fontana-Masson stain, which demonstrates melanin pigment, showed an absence of staining that began medial to the collarette, included the collarette, and extended laterally to the collarette. However, the MART-1 stain, which demonstrates melanocytes, showed preservation of melanocytes along the entire basal layer of the epidermis, including the areas of hypopigmentation.

Therefore, in the woman with halo lobular capillary hemangioma, the etiology for the loss of pigmentation surrounding the vascular lesion was associated with an acquired absence of melanin pigment with preservation of the melanocytes in the affected area. In contrast to vitiligo, in which the melanocytes would be diminished or absent in the location of depigmentation, the pathogenesis of halo formation associated with the lobular capillary hemangioma may be post-inflammatory; however, this lesion may be of prolonged duration such that not only the dermal stroma is fibrotic but the inflammatory infiltrate has completely resolved. Future reports of halo lobular capillary hemangioma may be helpful to either confirm our hypothesis for the development of the depigmented halo in the reported woman or establish an alternative mechanism of pathogenesis.

The term eruptive pseudoangiomatosis was coined in 1993; however, the condition was initially described as acute hemangioma-like lesions in children who had experienced a recent infection from either ECHO 25 virus or ECHO 32 virus. The condition was also observed in adults, and several other viruses have been associated with its development such as adenovirus, COVID-19, cytomegalovirus, Epstein-Barr virus, human immunodeficiency virus, and parvovirus B19. Other trigger factors associated with eruptive pseudoangiomatosis include acute lymphoblastic leukemia, chemotherapy, COVID-19 vaccines, Hodgkin lymphoma, insect bites, and non-Hodgkin lymphoma [18,19].

Eruptive pseudoangiomatosis presents clinically as small (1 to 4 millimeter) discrete cherry-angioma-like blanchable papules; the lesions are asymptomatic or slightly pruritic. A characteristic feature is that each vascular papule is surrounded by a white halo. The lesions occur on the face, neck, extremities, and trunk; in some of the patients, halos are not present on facial lesions [18,19].

The skin lesions are typically preceded by a prodrome of mild fever, sore throat, and/or gastrointestinal symptoms. They resolve spontaneously in about six days in children and 14 days in adults. If symptomatic, topical corticosteroids or oral antihistamines, or both, may be used [18,19].

The lesions are not usually biopsied. In a six-year-old boy, the lesion showed many dilated capillaries, lined by endothelial cells with a hobnail appearance, in the papillary and reticular dermis. The number of blood vessels was not increased; however, a mild-to-moderate perivascular lymphohistiocytic infiltrate was present [18,19].

Investigators have postulated that eruptive pseudoangiomatosis is either a viral exanthem (or an unusual reaction to other organisms) or a paraviral eruption to an antigen union at the vascular site. Some researchers considered the lesions to be similar to those of erythema punctatum Higuchi that have been noted in Japan and resulting from bites by Culex pipiens pallens. In addition to a viral exanthema, the clinical differential diagnosis includes Leukocytoclastic vasculitis and popular urticaria [18,19].

## Conclusions

The halo phenomenon refers to a hypopigmented or white halo surrounding a skin neoplasm. Cutaneous tumors with a perilesional halo include basal cell carcinoma, benign and malignant melanocytic neoplasms, seborrheic keratosis, surgical scars, and vascular lesions. A MART-1 stain was used to determine the presence or absence of melanocytes, and a Fontana-Masson stain was used to assess the expression of melanin pigment in the perilesional white and hypopigmented areas of a halo lobular capillary hemangioma. The epidermal basal layers of the entire specimen showed a normal number of uniformly distributed melanocytes, and the white collarette and hypopigmented halo revealed an absence of melanin pigment expression. In conclusion, halo lobular capillary hemangioma can be added to the congenital (capillary malformation-arteriovenous malformation and congenital hemangioma) and acquired (angioma, eruptive pseudoangiomatosis, and infantile hemangioma) benign vascular lesions that have been associated with halo phenomenon. A pathogenesis that involves post-inflammatory hypopigmentation may be responsible for the etiology of the halo lobular capillary hemangioma in the reported patient since the white epidermal collarette and hypopigmented perilesional halo revealed a loss of melanin pigment expression with the preservation of melanocytes.
